# Comparative Solid-State Stability of Perindopril Active Substance vs. Pharmaceutical Formulation

**DOI:** 10.3390/ijms18010164

**Published:** 2017-01-15

**Authors:** Valentina Buda, Minodora Andor, Adriana Ledeti, Ionut Ledeti, Gabriela Vlase, Titus Vlase, Carmen Cristescu, Mirela Voicu, Liana Suciu, Mirela Cleopatra Tomescu

**Affiliations:** 1 Faculty of Pharmacy, Victor Babes University of Medicine and Pharmacy, 2 Eftimie Murgu, 300041 Timisoara, Romania; buda.valentina.oana@gmail.com (V.B.); carmencristescu@umft.ro (C.C.); mavoicu@yahoo.com (M.V.); lianads@yahoo.com (L.S.); 2 Faculty of Medicine, Victor Babes University of Medicine and Pharmacy, 2 Eftimie Murgu, 300041 Timisoara, Romania; andorminodora@gmail.com (M.A.); tomescu.mirela@umft.ro (M.C.T.); 3 Research Centre for Thermal Analysis in Environmental Problems, West University of Timisoara, 300115 Timisoara, Romania; gabriela_vlase@e-uvt.ro (G.V.); titus_vlase@e-uvt.ro (T.V.)

**Keywords:** perindopril erbumine, perindopril *tert*-butylamine, thermal stability, decomposition, pharmaceutical formulation, comparative stability, isoconversional kinetic study, ASTM E698

## Abstract

This paper presents the results obtained after studying the thermal stability and decomposition kinetics of perindopril erbumine as a pure active pharmaceutical ingredient as well as a solid pharmaceutical formulation containing the same active pharmaceutical ingredient (API). Since no data were found in the literature regarding the spectroscopic description, thermal behavior, or decomposition kinetics of perindopril, our goal was the evaluation of the compatibility of this antihypertensive agent with the excipients in the tablet under ambient conditions and to study the effect of thermal treatment on the stability of perindopril erbumine. ATR-FTIR (Attenuated Total Reflectance Fourier Transform Infrared) spectroscopy, thermal analysis (thermogravimetric mass curve (TG—thermogravimetry), derivative thermogravimetric mass curve (DTG), and heat flow (HF)) and model-free kinetics were chosen as investigational tools. Since thermal behavior is a simplistic approach in evaluating the thermal stability of pharmaceuticals, in-depth kinetic studies were carried out by classical kinetic methods (Kissinger and ASTM E698) and later with the isoconversional methods of Friedman, Kissinger-Akahira-Sunose and Flynn-Wall-Ozawa. It was shown that the main thermal degradation step of perindopril erbumine is characterized by activation energy between 59 and 69 kJ/mol (depending on the method used), while for the tablet, the values were around 170 kJ/mol. The used excipients (anhydrous colloidal silica, microcrystalline cellulose, lactose, and magnesium stearate) should be used in newly-developed generic solid pharmaceutical formulations, since they contribute to an increased thermal stability of perindopril erbumine.

## 1. Introduction

Angiotensin-converting enzyme (ACE) inhibitors are a group of therapeutic agents widely used and listed as first-line agents in the treatment of hypertension, congestive heart failure, myocardial infarction, and left ventricular systolic dysfunction; they are either used alone or in combination with other classes of drugs with different mechanisms of action [1,2,3,4]. All the positive effects on the pathologies mentioned previously are the consequence of their mechanism of action: due to the blocking of ACE (angiotensin-converting enzyme), they decrease the formation of angiotensin II and the degradation of bradykinin [4].

Several ACE inhibitors are currently available on the market and they differ in their chemical structure of their active moieties, bioavailability, distribution, whether they are administered as prodrugs or not, plasma half-life, affinity for tissue-bond, etc. [5]. Perindopril, lisinopril and enalapril, have a carboxyl active moiety, captopril and zofenopril have a sulfhydryl group, and fosinopril is the only ACE inhibitor containing a phosphinyl group as an active moiety [5]. The majority of substances of the class are prodrugs, except captopril and lisinopril.

Perindopril (PER) is a third generation ACE inhibitor that was firstly developed in the 1980s for lowering blood pressure. Currently, PER is one of the most used and most studied API (active pharmaceutical ingredient) from its class, due to his excellent and favorable properties in decreasing not only the elevated blood pressure, but also the mortality and morbidity of cardiovascular disorders and by offering cardiovascular and renal protection [1,4].

Among the ACE inhibitors available, PER is the most used because of the advantages it possesses: high bioavailability; high terminal elimination half-life of the major active ingredient; high time to reach maximum plasma concentration [5]; strong ACE inhibition (the active metabolite of perindopril, perindoprilat, inhibits ACE activity in a greater way than enalaprilat, the active metabolite of enalapril) [6]; high lipophilicity and tissue penetration [7], prolonged duration of action [6]; and prolonged inhibition of ACE (>48 h) [8]. Also, at the onset, the pharmacodynamic effects of PER are slower but more sustained, and this slow onset of action contributes to a reduced risk of a first-dose hypotension [5] and 24-h efficacy which in turn contributes to a lower number of administrations per day and a higher compliance of patients for the treatment [5].

In the last 20 years, several studies (mostly large trials) revealed PER efficacy in the following: reduction of hypertension, as a long-acting, effective, well-tolerated antihypertensive agent [3,9,10]; reduction of left ventricular hypertrophy and all causes of cardiovascular mortality and morbidity [3,11,12]; reduction of the risk of stroke (PROGRESS study) [13]; prevention of cardiovascular remodeling and reduction of mortality and morbidity after myocardial infarction (EUROPA study) [14], after which EMA and FDA extended perindopril’s indications to include secondary prevention in CAD (coronary artery disease) patients; improvement of endothelial function and reduction of endothelial damage caused by cardiovascular risk factors (PERTINENT study and others) [15,16,17,18]; reduction of diabetic nephropathy and neuropathy (ADVANCE study) [19]; and positive influence on cognitive function [20].

Although it is a well-tolerated ACE inhibitor, PER shares the common side effects of the class but with a lower risk of incidence; it can cause dry cough (due, at least partially, to the accumulation of bradykinines), hypotension, hyperkalemia (because of a decrease in aldosterone), and a reversible decline in renal function (due to decreased renal perfusion secondary to bilateral renal artery stenosis, volume depletion, or severe congestive heart failure) [3].

PER is currently available on the market in the form of tablets for oral administration. The tablets contain one of the two types of salts available: perindopril *tert*-butylamine (or erbumine) of 2, 4, or 8 mg strengths or perindopril arginine of 5 or 10 mg. The *tert*-butylamine salt has a shelf life of about 2 years in countries with a temperate climate, and it requires special packaging conditions for countries with higher temperatures and humidity. A new salt, perindopril arginine, has been developed in order to improve the stability and shelf life of this drug. The doses of 5–10 mg of perindopril arginine are bioequivalent to 4–8 mg of perindopril *tert*-butylamine, but the arginine salt is 50% more stable and it has a shelf life of 3 years [3].

**Figure 1:**
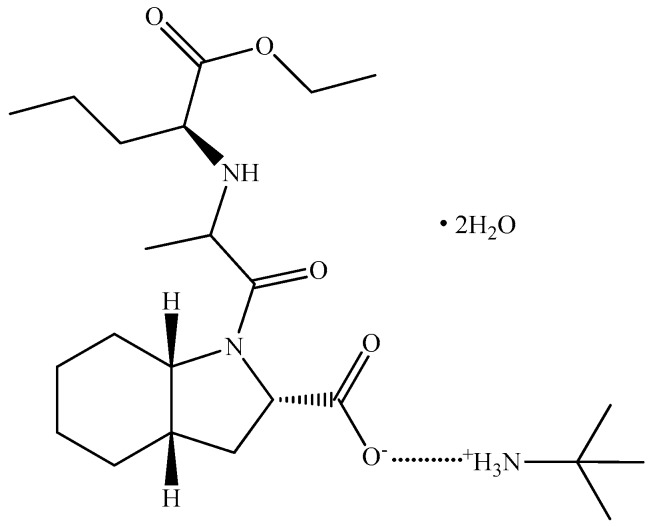
Structural formula of PER (perindopril erbumine dihydrate). The dotted line between the carboxylate moiety and the charged amine suggest the H-bonding interaction in the formation of the binary adduct.

Even if the stability of PER formulated as *tert*-butylamine salts seems to be lower than that of the one containing arginine, it is widely used in most solid formulations, including generics. The structural formula of perindopril erbumine dihydrate (PER) is presented in Figure 1.

Literature data published in the field of solid-state characterization of PER is rather poor. Gumieniczek et al. [21] studied the dissolution profile of PER, while Dorniani et al. [22] reported the preparation and characterization of magnetic nanoparticles coated with chitosan-perindopril erbumine, including some data regarding their thermal stability and dissolution profile. Kinetic studies were carried out solely regarding the isomerization of PER in the condensed phase using dynamics chromatography [23], and the two degradation pathways in aqueous solutions [24]. The studies of Rahman et al. [25] aimed at obtaining a sensitive kinetic spectrophotometric method for the determination of PER in pharmaceutical preparations. As contributions in the field of evaluation of the thermal stability of several antihypertensive drugs, like captopril, nifedipine, and propanolol hydrochloride, Macedo et al. [26] published a study regarding the decomposition kinetics by using two methods: Coats-Redfern, and Madhusudanan, respectively. In more recent papers, thermal behavior of the β-blocker anti-hypertensive drug propranolol was investigated using thermoanalytical techniques, providing information regarding thermal stability and decomposition steps [27], and the thermal stability and decomposition of amlodipine besylate was reported [28].

Since some literature references were found for the physico-chemical characterization of perindopril, including polymorphism, solvatomorphism, and thermal stability in inert atmosphere [29], but no data was found regarding thermal stability and decomposition kinetics of perindopril in air, we designed this study to investigate the solid-state stability of perindopril *tert*-butylamine (PER_as_) as pure API vs. a commonly-used generic tablet containing the same API (PER_pf_). ATR-FTIR (Attenuated Total Reflectance Fourier Transform Infrared) spectroscopy was used to obtain information about the compatibility of the API with the excipients used in the solid formulation, followed by a search of the interactions between the components during thermal treatment. As the most reliable tool regarding the comparative stability of PER_as_ vs. PER_pf_, a complete kinetic study was preliminary carried out by the employment of Kissinger and ASTM E698 methods, and later completed by model-free methods which include the isoconversional methods of Friedman (Fr), Flynn-Wall-Ozawa (FWO) and Kissinger-Akahira-Sunose (KAS).

## 2. Results

In this study, three investigational methods were employed in order to characterize the behavior of PER_as_ in comparison with a solid pharmaceutical formulation containing the maximum amount of PER per tablet (8/100 mg tablet). The strength was chosen in order to maximize the effect of observable interactions between the tablet component during thermal treatment, and to aid in the correct identification of thermal events associated with thermolysis of the active pharmaceutical ingredient in the presence of excipients.

### 2.1. ATR-FTIR Investigations

**Figure 2:**
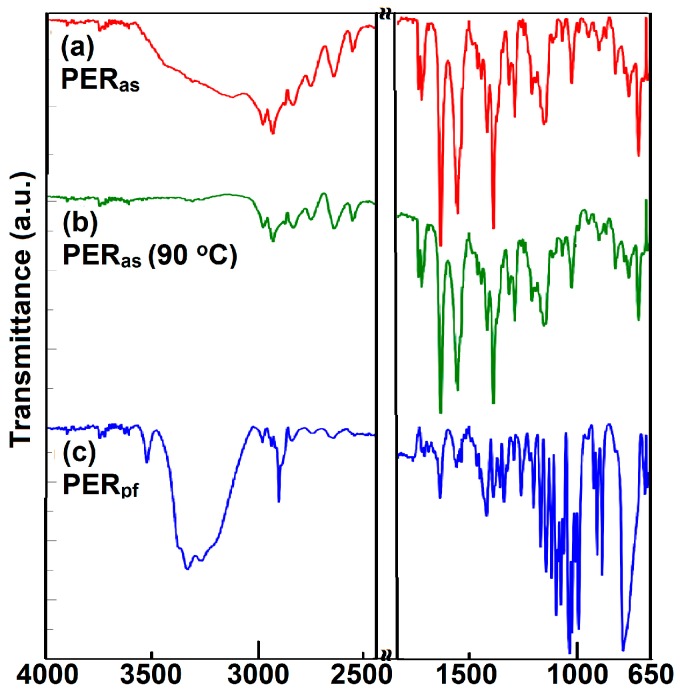
ATR-FTIR spectra recorded on spectra range 4000–650 cm^−1^ in solid state for: (**a**) perindopril *tert*-butylamine as the pure active pharmaceutical ingredient (PER_as)_; (**b**) PER_as_ kept for 24 h in isothermal conditions at 90 °C and (**c**) a commonly-used generic tablet containing the same active pharmaceutical ingredient (API) as PER_as_ (PER_pf_). The spectral range 2400–2000 cm^−1^ was suppressed due to the presence of ATR background bands.

In order to confirm the purity of pure PER (abbreviated PER_as_), ATR-FTIR was chosen as the spectroscopic investigation tool. The spectra were determined and compared to the pharmaceutical formulation (PER_pf_) in order to identify if any modification of the API occur during preparation of the final pharmaceutical formulation. The comparative obtained spectra are presented in Figure 2a,b.

### 2.2. Thermal Stability Investigations

**Figure 3:**
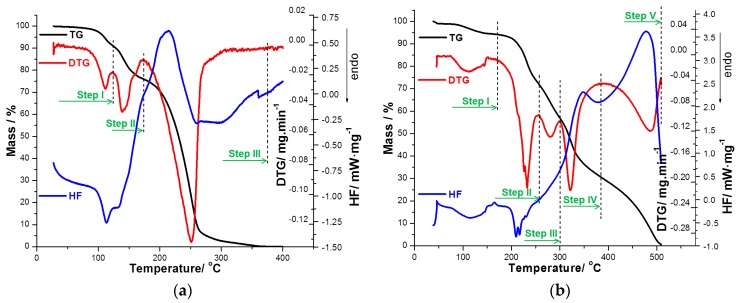
Simultaneously-determined TG (thermogravimetric mass curve), DTG (derivative thermogravimetric mass curve), and normalized HF (heat flow) curves in oxidative air atmosphere at β = 5 °C·min^−1^ for: (**a**) PER_as_ in the temperature range of 40–400 °C and (**b**) PER_pf_ in the temperature range of 40–500 °C.

A comparative thermal stability of PER_as_ and PER_pf_ was carried out in an oxidative atmosphere at a heating rate of 5 °C·min^−1^, as shown in Figure 3a,b.

For each sample, thermogravimetric mass curve (TG), derivative thermogravimetric mass curve (DTG), and normalized heat flow (HF) data were recorded in identical experimental conditions in order to obtain comparable experimental data. The two analyzed samples have different thermal behavior due to the different composition, but in the case of PER_pf_, the characteristic pattern for the active substance was identified. The decomposition begins at the same temperature for each sample, but the degradation mechanism is different as identified by a different number of steps, namely three steps for PER_as_ and five for PER_pf_. The difference appeared due to the presence of excipients in a considerable amount in the pharmaceutical formulation.

### 2.3. Kinetic Study

The kinetic study was performed using the DTG data obtained in air atmosphere for the decomposition of PER_as_ and PER_pf_ samples at five heating rates: β = 5, 7, 10, 12, and 15 °C·min^−1^. The preliminary kinetic study was carried out using the Kissinger and ASTM E698 methods.

**Figure 4:**
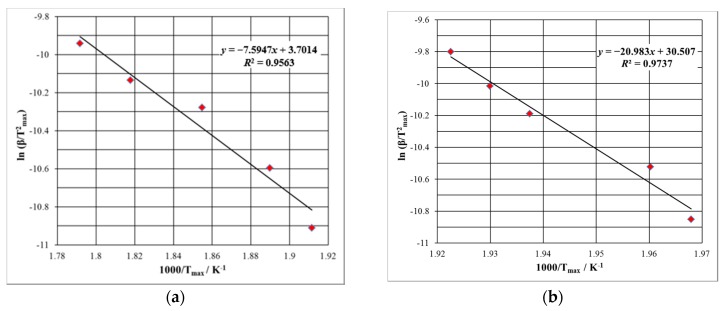
Kissinger kinetic method linear plottings for PER_as_ (**a**) and PER_pf_ (**b**).

A first evaluation for the kinetic decomposition was realized by the use of the Kissinger method, which states that for an Arrhenius-type dependence of the rate constant vs. temperature, a mathematical model represented by Equation (1) can be obtained:

(1) $$\ln(\beta \cdot T_{\max}^{-2})=\ln(A\cdot R\cdot E_{a}^{-1})+\ln[n\cdot {\left(1-\alpha_{\max}\right)}^{n-1}]-E_{a}\cdot R^{-1}\cdot T_{\max}^{-1}$$

where *E*_a_ is the activation energy, *A* is the pre-exponential factor, β is the heating rate, *n* is the reaction order, α is the conversion degree, *T* is the absolute temperature, R is the gas constant, and index max is used for indicating the maximum of the reaction rate. As 1 − α_max_ is constant for a certain value of *n*, the evaluation of the *E*_a_ can be achieved by determining the slope of the linear plotting of ln (β*·*$T_{max}^{-2}$) vs. 1000/*T*_max_ for experiments carried out at different heating rates [30], as shown in Figure 4a,b.

As second preliminary method, ASTM E698 kinetic method was used. This method is based on the Ozawa plot, which is based on the assumption that the degree of reaction is a constant value independent of the heating rate when a DTG curve reaches its peak (Equation (2)):

(2) $$\ln\beta =const-1.052\cdot E_{a}\cdot R^{-1}\cdot T_{\max}^{-1}$$

**Figure 5:**
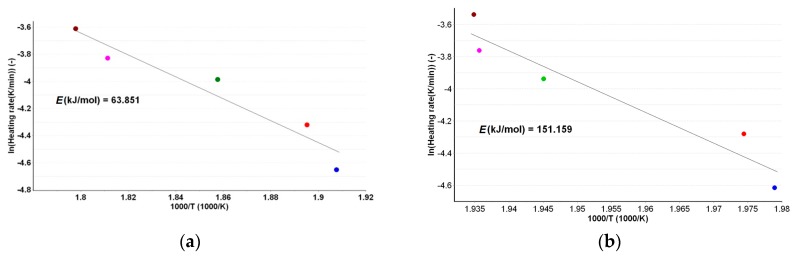
ASTM E698 kinetic method linear plottings for PER_as_ (**a**) and PER_pf_ (**b**). The different colored dots represent the DTG_peaks_ at different heating rates.

The evaluation of the *E*_a_ can be achieved by evaluating the slope of the linear plottings for experiments carried out at the five different heating rates (Figure 5a,b).

The activation energies obtained by using Kissinger and ASTM E698 methods are presented in Table 1.

**Figure 6:**
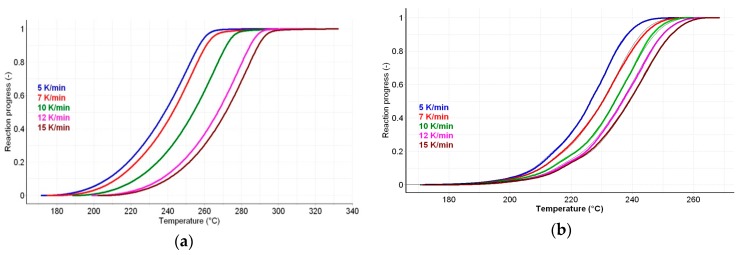
The progress of reaction vs. temperature for PER_as_ (**a**) and PER_pf_ (**b**).

However, ICTAC 2000 recommendations indicate the use of isoconversional methods. The advantage of using thermal analysis and a kinetic study was extensively described previously [31,32,33,34,35,36,37,38,39,40,41]. Three isoconversional methods, a differential one (Friedman) and two integral ones (Flynn-Wall-Ozawa and Kissinger-Akahira-Sunose), were employed in order to determine the values of *E*_a_ vs. the conversion degree α. The progress of reaction vs. temperature is presented in Figure 6a,b, for the active substance and the pharmaceutical formulation.

The mathematical models of isoconversional methods as well their deduction were reported earlier, elsewhere [42]. Briefly, these models are presented as follows.

Friedman method (Fr) [43] is used in the linearized form, as shown in Equation (3).

ln (β × dα/d*T*) = ln [*A* × *f*(α)] − *E*_a_*·*R*^−1^·T^−1^* (3)

**Figure 7:**
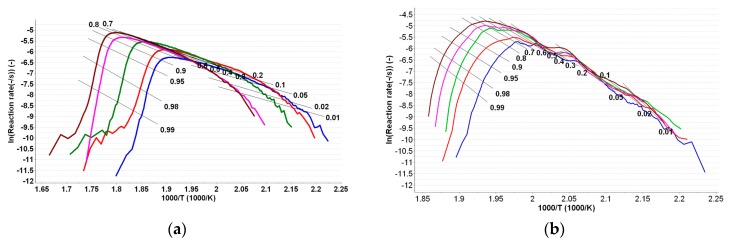
Linear plotting of Friedman method at selected heating rates for PER_as_ (**a**) and PER_pf_ (**b**).

For known α at the selected heating rates, the plot ln (β × dα/d*T*) vs. (1/*T*) is linear. Evaluating the slopes of these straight lines (see Figure 7a,b), the values of the activation energy (*E*_a_) for the two samples are obtained (Table 2).

The isoconversional Flynn-Wall-Ozawa (FWO) method [44,45] is used in the following linearized form (Equation (4)):

ln β = ln [*A × E·R*^−1^·g^−1^(α)] − 5.331 − 1.052·*E*_a_*·*R^−1^·*T*^−1^ (4)

where g(α) is the integral conversion function.

**Figure 8:**
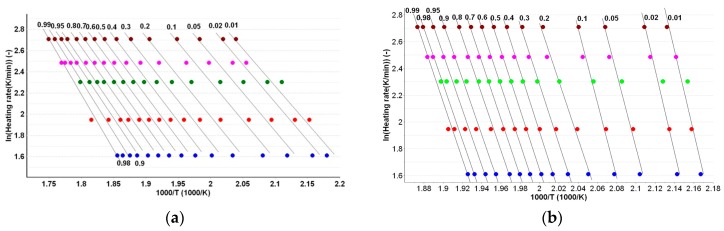
Linear plotting of Flynn-Wall-Ozawa method at selected heating rates for PER_as_ (**a**) and PER_pf_ (**b**). The different colored dots represent the DTG_peaks_ at different heating rates.

The plotting of ln β vs. *T*^−1^ allows the estimation of activation energy values (*E*_a_) for all the conversion degrees, which are presented in Figure 8a,b and in Table 2.

Kissinger, Akahira and Sunose developed an integral isoconversional method (KAS) [46,47], generally used in the linearized form shown by Equation (5).

ln (β × *T^−2^*) = ln [*A*·*R·E*_a_^−1^·*g*^−1^(α)] – *E*_a_*·*R*^−1^*·*T*^−1^ (5)

**Figure 9:**
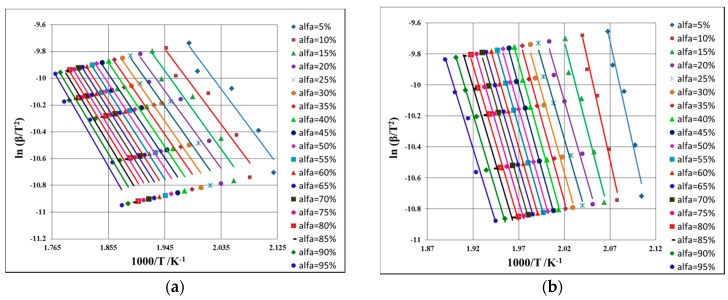
Linear plotting of Kissinger-Akahira-Sunose method at selected heating rates for PER_as_ (**a**) and PER_pf_ (**b**).

A similar protocol for evaluation of *E*_a_ at each *α* like in the case of the FWO method is used, with the difference of plotting of ln β × *T*^−2^ vs. *T*^−1^ (see Figure 9).

## 3. Discussion

### 3.1. ATR-FTIR Investigations

The ATR-FTIR technique was chosen over classical KBr dispersion, since the sample preparation, including pressure, pelleting can affect the stability of the API and induce interactions between the API and excipients. According to this consideration, the samples were investigated in solid state as received (PER_as_) or after crushing and pulverization with a pestle in an agate mortar and then sieved (PER_pf_).

ATR-FTIR spectra were comparatively recorded, in identical conditions, for PER_as_, PER_pf_, and for a PER_as_ sample maintained for 24 h in isothermal conditions at 90 °C (PER_as_ 90 °C). Since initial ATR-FTIR spectra of PER_as_ showed a broad band in the spectral range 3580–2460 cm^−1^, which was not reported in the paper of Dorniani et al. [22], some investigations were carried out in order to identify the nature of this broad band. A patent of Rucman and Zupet mentions the formation of hydrated crystalline forms of perindopril erbumine as monohydrate, sesquihydrate, and dihydrate [48], but ATR-FTIR spectra of these crystalline samples were not reported in the literature. Corroborating this spectroscopic information with the information suggested by thermal analysis (see Section 2.2 and Section 3.2) where the first mass loss occurs in the 82–124 °C temperature range, it was proven that dehydration occurs. A sample or pure PER_as_ was kept in isothermic conditions for 24 h at 90 °C and afterwards the ATR-FTIR spectrum was recorded. It was revealed that in the case of the thermally-treated sample, the 3580–2460 cm^−1^ broad band was no longer visible, confirming the dehydration. In Section 3.2, we present the discussion confirming that the amount of loss water corresponds to 2 mol per mol of PER, confirming the dihydrate crystalline form of PER_as_.

Otherwise, the ATR-FTIR spectra of PER_as_ and PER_as_ (90 °C) are practically identical, confirming that during thermal treatment at 90 °C for 24 h, thermolysis of salt does not take place, but only water removal occurs.

The ATR-FTIR spectra of PER_as_ (Figure 2a) reveal the presence of the functional bands contained in the molecular structure of the antihypertensive agent. The C–H stretching vibrations from the –CH_3_, CH_2_, and CH groups appear in the spectral range 2980–2800 cm^−1^ with peaks at 2974, 2928, and 2834 cm^−1^, respectively, and correspond to the presence of these groups in the structure of PER and the salt conformer—*tert*-butylamine. The methylene stretching vibrations of the hexacyclic methylene structure appear near the ones observed for linear alkanes. Cyclization decreases the frequency of the methylene scissoring with bands being observed in the 1466–1448 cm^−1^ spectral range. The N-H stretch from ammonium salt show combination bands in the 1800–1745 cm^−1^ spectra range, with peaks at 1772 and 1745 cm^−1^. Also, the N-H stretching vibrations for salts of the primary amine show multiple combination bands in the 2800–2500 cm^−1^ region, with peaks at 2749, 2641, and 2551 cm^−1^. The most intense and easily-observed bands are the ones due to the presence of carbonyl moieties, with peaks at 1731, 1640, 1561, and 1390 cm^−1^. These bands confirm the existence of the carboxylate anion due to salt formation with *tert*-butyl amine, by giving rise to two different bands: the asymmetrical stretching band at 1640 cm^−1^ and a weaker symmetrical stretching at 1390 cm^−1^. The C=O stretching vibration from substituted amide and ester moieties appear at 1731 and 1561 cm^−1^, respectively. The C–O stretching vibrations for the ester group consist in two asymmetrical coupled vibrations, in the spectra region 1300–1000 cm^−1^, as medium-intense bands with peaks at 1290 and 1152 cm^−1^. Skeletal vibrations for the cyclohexane ring appear in the fingerprint region, near 750 cm^−1^, while the skeletal vibration of the –C(CH_3_)_3_ moiety appear around 1210 cm^−1^ as a medium-intensity band. Other bands are hard to attribute due to the complex structure of PER; most of them are combination bands. The same bands are observed in the case of thermally-treated PER_as_ at 90 °C, being unshifted or shifted to ± 2 cm^−1^ (Figure 2b). The peaks (without attribution) identified in the spectrum of PER_as_ are (in cm^−1^): 2974, 2928, 2834, 2749, 2641, 2551, 1772, 1745, 1731, 1640, 1561, 1466, 1448, 1421, 1390, 1317, 1290, 1247, 1291, 1209, 1152, 1064, 1021, 988, 941, 891, 857, 813, 770, 750, and 704. These bands are presented here in order to identify them in the spectrum of PER_pf_ for evaluation of possible interactions between API and excipients.

The ATR-FTIR spectrum of PER_pf_ (Figure 2c) show a more complex pattern due to overlapping of characteristic bands of API and the ones of excipients including magnesium stearate, anhydrous colloidal silica, microcrystalline cellulose, and lactose. The characteristic bands of PER were identified at (in cm^−1^): 2977, 2931, 2838, 2748, 2642, 2552, 1772, 1747, 1730, 1643, 1565, 1469, 1447, 1424, 1391, 1325, 1295, 1295, 1202, 1019, 988, and 942. The bands are greatly attenuated in comparison to the ones of pure PER_as_ due to the diluting effect of excipients, especially the strong absorption bands of magnesium stearate and silica. However, the identified bands confirm the presence of API in the pharmaceutical formulation and the compatibility of PER with excipients under ambient conditions, which were further investigated by thermal analysis in order to determine if there are any thermally-induced interactions.

### 3.2. Thermal Stability Investigations

The thermoanalytical curves TG/DTG and normalized HF (Figure 3a,b) for the two samples PER**_as_** and the pharmaceutical form (PER_pf_) were investigated. These curves were recorded using a heating rate β = 5 °C·min^−1^, up to an approximately complete mass loss. Even if the two samples contain the same active substance, the thermal profiles are different, the cause being the presence of the excipients in appreciable quantities in the case of the pharmaceutical form.

According with the normalized HF curve (which can be referred to as the DSC curve) of PER_as_, a broad endothermic peak with a shoulder was observed with maximuma at 113.2 and 132.3 °C, respectively. The two maximums are accompanied by two well defined processes on the DTG curve (DTG_max_ at 109.7 and 138.6 °C) which define two mass loss processes: Δ*m*_1*experim*_ = 8.1% (DTG_onset_ = 80 °C) and Δ*m*_2*experim*_ = 15.54% (DTG_onset_ = 125 °C). The first mass loss (Step I) corresponds to the dehydration step of the dihydrated active substance Δ*m*_1*theoretical*_ 7.54% = 2·M_H2O_·100/M_perindopril tert-butyl-amine dihydrate_ (where m is the mass and M is molar mass). The second mass loss (Step II) has an appreciable extent and characterizes the loss of the salt coformer, i.e., *tert*-butylamine, which has a considerable volatility Δ*m_2theoretical_* = 15.31% = M_tert-butyl-amine_·100/M_perindopril tert-butyl-amine dihydrate_. Since a good agreement was found between the theoretically calculated water and coformer (erbumine) content, the active pharmaceutical ingredient was confirmed to be perindopril erbumine hydrate. The mass loss found in these two temperature ranges is practically equal with the calculated water and amine content; for each mol of perindopril, one mol of erbumine and two mol of water was determined. Practically, after 170 °C, all the thermal events (Step III) are due to thermal decomposition of PER, this being the reason why the kinetic study was carried out for the process between 170 and 320 °C. This mass loss is accompanied by an exothermal event with a maximum at 213 °C. The obtained thermal data are in partial agreement with the ones reported by Dorniani et al. [22], which interpreted incorrectly the DTG curve and reported the melting of PER by using the mass derivative curve, instead of the HF, DTA, or DSC profile. Also, the study of Dorniani et al. [22] suggested that perindopril contains surface-adsorbed water, but the correlation of our results with a previously published study for polymorphic and solvatomoporhic forms of PER [29] reveal that the calculated and found water content is due to crystallization water, in agreement with data reported by Rucman and Zupet [48]. However, the thermolysis is correctly reported in the above-mentioned paper [22], finally leading to the complete destruction of the molecular structure of PER [22].

The thermal decomposition of the pharmaceutical form PER_pf_ exhibits five successive and overlapping mass loss steps (Figure 3b) attributed to a total degradation of the pharmaceutical mixture, with a complete mass loss near 500 °C. The thermogravimetric curve reveals a water loss process from 80 to 170 °C, due to the dehydration of API (Step I), loss of *tert*-butylamine and dehydration/degradation of lactose, magnesium stearate, and microcrystalline cellulose [36]. After that, a new process is revealed by the DTG curve (Step II), namely the thermal decomposition of the API. This process is similar to the process seen on DTG curve of the active substance and for this process the kinetic parameters were also estimated.

The last three processes (Steps III–V) are attributed to the advanced degradation of the excipients (in pure phase, according at DTG curve, lactose presents a thermal decomposition with a maximum at 300 °C, microcrystalline cellulose at 325 °C, and magnesium stearate at 250 and 420 °C) [36]. These events lead to a complete destruction of all organic skeletons. The summarized results obtained after carrying out the thermal analysis are presented in Table 3.

However, a tentative conclusion regarding the compatibility of the API under thermal treatment with the excipients in the solid formulation cannot be drawn, since the normalized HF curve does not indicate a clear and well-defined event of melting of the API (i.e., solid-liquid transition), and in the same temperature range, the degradation of excipients also occurs in overlapping events.

### 3.3. Kinetic Study

The kinetic methods of Kissinger and ASTM E698 indicate a similar stability in terms of apparent activation energy, since the estimated values are similar (Table 1). However, the values do not indicate a very good thermal stability of PER_as_, since the value is around 63 kJ/mol. The same protocols applied for the degradative process of PER_pf_ lead to a considerably higher value for activation energy, i.e., around 150 kJ/mol by ASTM E698, and 174 kJ/mol by the Kissinger method. There are two possible explanations, namely: the stabilizing effect of excipients over degradation of the API, or the superimposing of decomposition steps of excipients over API. The existence of parallel degradative processes may also be suggested by the ASTM E698 method, since the R^2^ value is not indicating a clear linear dependency (*R*^2^ = 0.92 for PER_as_ and 0.93 for PER_pf_).

Due to these inadvertencies, an isoconversional kinetic study was carried out. The main advantage of an isoconversional kinetic study is that the estimation of *E*_a_ values for each conversion degree can lead to appreciation if the degradative mechanism is dependent of heating rate. Since the degradative mechanism is not known for the thermolysis of most pharmaceuticals, another advantage of isoconversional methods is that they allow for the evaluation of activation energy without knowing the explicit form of the differential or integral conversion function.

It is generally considered that the evaluation of apparent activation energy for each conversion degree can lead to information regarding the single-step or multistep degradation of a pharmaceutical compound [39]. If the *E*_a_ vs. α values are estimated between ± 10% around the medium value of *E*_a_, the degradative mechanism consists of a single-step process, which is invariable with the modification of the heating rate of the sample (i.e., an independent mechanism with increase or decrease of heating rate). For multi-step parallel or successive degradative processes, the estimated apparent activation energies fall outside of the ± 10% interval, and in this case, the compound follows different decomposition pathways as the reaction advances and is dependent on the heating rate of the sample. Isoconversional methods are powerful tools in indicating the single-step or multi-step mechanism process for degradation of the API. Following these considerations, each isoconversional method is discussed below.

The Friedman method indicated a variation outside the 10% limit around the medium value of *E*_a_, for PER_as_ and PER_pf_, especially at lower and higher conversions. For PER_as_, a variation of ± 10 kJ/mol was observed for α = 5% and α > 90%. In the case of PER_pf_, the variation was more irregular, clearly suggesting the complex pathway of degradation for the mixture of API with excipients.

In the case of integral isoconversional methods, the variation of *E*_a_ vs. α was less variable including at extreme conversion degrees. This fact can be explained by the integral processing of the data, in comparison to the differential processing of the Friedman method.

Both kinetic studies (classic methods of Kissinger and ASTM E698 and isoconversional ones) suggested an increased stability in terms of apparent activation energy of the pharmaceutical formulation in comparison to pure API, leading to the conclusion that the used excipients can be utilized in future generic forms, leading to highly-stable formulations.

## 4. Materials and Methods

### 4.1. Samples and Preparation

Perindopril tert-butylamine CRS—catalogue reference standard (PER, Batch 2.1, Id 009MV4) according to the European Pharmacopoeia Reference Standard was a commercial product from the European Directorate for the Quality of Medicines & Healthcare EDQM, Council of Europe (Strasbourg, France) and was used without further purification.

The pharmaceutical formulation was a generic tablet with a strength of 8 mg PER, which is the highest strength usually available. The tablet was crushed in an agate mortar with a pestle, homogenized for five minutes, and then sieved. As excipients, the manufacturer declared the presence of anhydrous colloidal silica, microcrystalline cellulose, lactose, and magnesium stearate.

### 4.2. Spectroscopic Investigations

ATR-FTIR spectra were recorded on a Perkin Elmer SPECTRUM 100 device (Perkin-Elmer Applied Biosystems, Foster City, CA, USA), without a priori preparation of the sample. The data was collected in a 4000–650 cm^−1^ domain, on an UATR device. Spectra were built up after a number of 32 co-added scans.

### 4.3. Thermal Stability Investigations

Thermal analysis investigations were carried out on a Perkin-Elmer DIAMOND apparatus (Perkin-Elmer Applied Biosystems, Foster City, CA, USA) for obtaining simultaneously the TG (thermogravimetric/mass curve), DTG (derivative thermogravimetric/mass derivative) and HF (heat flow) in dynamic air atmosphere (100 mL·min^−1^), using aluminum crucibles. The analyses were carried out under non-isothermal conditions at five heating rates, β, namely 5, 7, 10, 12, and 15 °C·min^−1^ from ambient up to 400/500 °C. For determining the thermal effects, the DTA data (in µV) were converted to HF (Heat Flow) data (mW). The HF data (mW) were converted to normalized HF data by dividing the signal by mass of sample, obtaining the DSC data (in mW·mg^−1^).

In order to assure the reproducibility of the TG study, each analysis was repeated three times and the results were comparable.

### 4.4. Kinetic Study

The kinetic study (Friedman, Flynn-Wall-Ozawa, and ASTM E698 methods) was carried out on the main decomposition step that took place between 180–320 °C using the AKTS—Thermokinetics Software (AKTS AG TechnoArk, Siders, Switzerland). Kissinger and Kissinger-Akahira-Sunose methods were applied using a template developed by our group. All the mathematical background and importance of using isoconversional kinetic methods is extensively reported in literature [30,39,40,43,44,45].

## 5. Conclusions

This paper presented a comparative study regarding the spectroscopic description, thermal stability, and evaluation of decomposition kinetics for perindopril erbumine as a pure active pharmaceutical ingredient and as when formulated in a solid pharmaceutical form. ATR-FTIR spectroscopy revealed that under ambient conditions, perindopril erbumine is compatible with anhydrous colloidal silica, microcrystalline cellulose, lactose, and magnesium stearate, since the main bands observed in the case of pure API are presented in the spectrum of the mixture. Thermal analysis revealed the presence of thermally-induced interactions and a modification of the degradative pathway of the API, but the temperature of occurring interactions could not be evaluated since the degradation of API is superposed over the degradation of excipients.

In order to evaluate the excipient effect over the solid-state decomposition kinetics, the classical kinetic methods of Kissinger and ASTM E698 were used, and data were compared to the data obtained by isoconversional methods. It was shown that perindopril erbumine is characterized by a decomposition energy between 59 and 69 kJ/mol (depending the method used), while for the tablet, the values were around 170 kJ/mol.

The used excipients (anhydrous colloidal silica, microcrystalline cellulose, lactose, and magnesium stearate) should be used in newly-developed generic solid pharmaceutical formulations, since they contributed to an increased thermal stability of perindopril erbumine.

## Figures and Tables

**Table 1:** Activation energy values obtained by Kissinger and ASTM E698 methods.

Sample	*E*_a_ (kJ·mol^−1^)	
	Kissinger	ASTM E698
PER_as_	63.1	63.9
PER_pf_	174.5	151.2

**Table 2:** Evaluation of activation energy (*E*_a_) values vs. conversion degree obtained by the three isoconversional methods and the mean value of *E*_a_.

Conversion Degree α	*E*_a_ (kJ·mol^−1^) vs. α for PER_as_			*E*_a_ (kJ·mol^−1^) vs. α for PER_pf_		
	Fr	KAS	FWO	Fr	KAS	FWO
0.05	59.6	54.1	59.2	190.6	178.2	179.3
0.10	60.2	54.8	60.1	194.1	176.5	177.2
0.15	60.9	55.6	60.9	194.0	167.2	168.9
0.20	61.2	56.0	61.4	193.9	169.5	169.7
0.25	62.9	56.3	61.7	186.7	169.0	171.4
0.30	65.6	56.8	62.2	186.8	174.2	174.4
0.35	67.0	57.5	62.9	188.2	176.0	176.5
0.40	67.8	57.7	63.1	176.7	176.2	177.6
0.45	69.0	58.4	63.8	162.2	175.1	176.6
0.50	69.9	59.1	64.5	159.5	174.0	174.4
0.55	71.3	59.9	65.3	167.8	173.1	173.2
0.60	71.9	60.5	65.9	163.3	170.3	172.3
0.65	72.6	61.3	66.7	162.9	169.9	171.4
0.70	74. 1	61.9	67.3	153.4	168.5	169.8
0.75	74.8	62.6	68.0	153.1	166.1	167.8
0.80	76.0	63.4	68.8	165.0	166.6	166.7
0.85	77.9	64.2	69.6	162.7	164.7	166.2
0.90	79.3	65.2	70.6	155.9	164.9	164.7
0.95	79.8	65.7	71.1	164.9	159.6	163.6
$\bar{E_{a}}$ (kJ·mol^−1^)	69.3 ± 6.5	59.5 ± 3.5	64.9 ± 3.5	172.7 ± 14.7	170.5 ± 4.9	171.7 ± 4.5

**Table 3:** Results of parameters obtained from the analysis of TG-DTG and normalized HF curves.

Samples	Step	Temperature Range/°C	DTG_max_/°C	Normalized HF		Δ*m*/%
				*T*_onset_/°C	*T*_peak_/°C	
PER_as_	I	40.0–121.8	110.9	83.2	113.2	8.10
	II	121.8–166.9	137.4	151.2	132.3	15.54
	III	166.9–374.9	249.6	171.5	213.1	76.36
PER_pf_	I	40.0–171.2	108.9	70.0	110.5	5.81
	II	171.2–253.5	222.6; 232.3	165.8	204.7; 216.1	21.30
	III	253.5–298.9	277.8	-	-	15.60
	IV	298.9–381.7	320.0	310.3	342.8	26.20
	V	381.7–500.0	485.6	380.1	479.2	31.09

## References

[1] Hanif K., Bid K.H., Konwar R. (2010). Reinventing the ACE inhibitors: Some old and new implications of ACE inhibition. Hypertens. Res..

[2] Mancia G., Fagard R., Narkiewicz K., Redon J., Zanchetti A., Bohm M., Christiaens T., Cifkova R., de Backer G., Dominiczak A. (2013). Task force for the management of arterial hypertension of the European society of hypertension and the European society of cardiology. 2013 ESH/ESC guidelines for the management of arterial hypertension. Eur. Heart J..

[3] Fox K. (2007). Contribution of perindopril to cardiology: 20 years of success. Eur. Heart J..

[4] Tantu M., Belu E., Bobescu E., Armean S.M., Armean P., Constantin M.M., Dominaru C.D. (2014). Role of angiotensin converting enzyme (ACE) inhibitors in hypertension and cardiovascular protection management. Farmacia.

[5] Cockcroft J.R. (2007). ACE inhibition in hypertension: Focus on perindopril. Am. J. Cardiovasc. Drugs.

[6] Louis W.J., Conway E.L., Krum H., Workman B., Drummer O.H., Lam W., Phillips P., Howes L.G., Jackson B. (1992). Comparison of the pharmacokinetics and pharmacodynamics of perindopril, cilazapril and enalapril. Clin. Exp. Pharmacol. Physiol. Suppl..

[7] Ferrari R. (2005). Angiotensin-converting enzyme inhibition in cardiovascular disease evidence with perindopril. Expert Rev. Cardiovasc. Ther..

[8] Louis W.J., Workman B.S., Conway E.L., Worland P., Rowley K., Drummer O., McNeil J.J., Harris G., Jarrott B. (1992). Single-dose and steady-state pharmacokinetics and pharmacodynamics of perindopril in hypertensive subjects. J. Cardiovasc. Pharmacol..

[9] Guo W., Turlapaty P., Shen Y., Dong V., Batchelor A., Barlow D., Lagast H. (2004). Clinical experience with perindopril in patients nonresponsive to previous antihypertensive therapy: A large US community trial. Am. J. Ther..

[10] Julius S., Cohn J.N., Neutel J., Weber M., Turlapaty P., Shen Y., Dong V., Batchelor A., Lagast H. (2004). Antihypertensive utility of perindopril in a large, general practice-based clinical trial. J. Clin. Hypertens..

[11] London G.M., Pannier B., Guerin A.P., Marchais S.J., Safar M.E., Cuche J.L. (1994). Cardiac hypertrophy, aortic compliance, peripheral resistance, and wave reflection in end-stage renal disease. Comparative effects of ACE inhibition and calcium channel blockade. Circulation.

[12] Guerin A.P., Blacher J., Pannier B., Marchais S.J., Safar M.E., London G.M. (2001). Impact of aortic stiffness attenuation on survival of patients in endstage renal failure. Circulation.

[13] PROGRESS Collaborative Group (2001). Randomised trial of a perindoprilbased blood-pressure-lowering regimen among 6105 individuals with previous stroke or transient ischaemic attack. Lancet.

[14] Fox K.M. (2003). Efficacy of perindopril in reduction of cardiovascular events among patients with stable coronary artery disease: Randomised, double-blind, placebo-controlled, multicentre trial (the EUROPA study). Lancet.

[15] Ceconi C., Fox K.M., Remme W.J., Simoons M.L., Bertrand M., Parrinello G., Kluft C., Blann A., Cokkinos D., Ferrari R. (2007). EUROPA Investigators; PERTINENT Investigators and the Statistical Committee. ACE inhibition with perindopril and endothelial function. Results of a substudy of the EUROPA study: PERTINENT. Cardiovasc. Res..

[16] Buda V., Andor M., Cristescu C., Voicu M., Suciu L., Suciu M., Tomescu M. (2014). Blockers of the RAA system: Perindopril and candesartan and their implication on endothelial dysfunction. Med. Evol..

[17] Buda V., Tomescu M., Cristescu C. (2014). The relationship between the bradykinins, RAAS and ACE inhibitors: An overview. Med. Evol..

[18] Buda V., Andor M., Cristescu C., Voicu M., Suciu L., Muntean C., Cretu O., Baibata D.E., Gheorghiu C.M., Tomescu M.C. (2016). The influence of perindopril on PTX3 plasma levels in hypertensive patients with endothelial dysfunction. Farmacia.

[19] Patel A., MacMahon S., Chalmers J., Neal B., Woodward M., Billot L., Harrap S., Poulter N., Marre M., ADVANCE Collaborative Group (2007). Effects of a fixed combination of perindopril and indapamide on macrovascular and microvascular outcomes in patients with type 2 diabetes mellitus (the ADVANCE trial): A randomised controlled trial. Lancet.

[20] Amenta F., Mignini F., Rabbia F., Tomassoni D., Veglio F. (2002). Protective effect of antihypertensive treatment on cognitive function in essential hypertension: Analysis of published clinical data. J. Neurol. Sci..

[21] Gumieniczek A., Maczka P., Komsta L., Pietras R. (2015). Dissolution profiles of perindopril and indapamide in their fixed-dose formulations by a new HPLC method and different mathematical approaches. Acta Pharm..

[22] Dorniani D., Hussein M.Z.B., Kura A.U., Fakurazi S., Shaari A.H., Ahmad Z. (2013). Sustained release of prindopril erbumine from its chitosan-coated magnetic nanoparticles for biomedical applications. Int. J. Mol. Sci..

[23] Bouabdallah S., Trabelsi H., Ben Dhia M.T., Ben Hamida N. (2012). Kinetic Study on the Isomerization of Perindopril by HPLC. Chromatographia.

[24] Simoncic Z., Rokar R., Gartner A., Kogej K., Kmetec V. (2008). The use of microcalorimetry and HPLC for the determination of degradation kinetics and thermodynamic parameters of Perindopril Erbumine in aqueous solutions. Int. J. Pharm..

[25] Rahman N., Anwar N., Kashif M. (2006). Optimized and validated initial-rate method for the determination of perindopril erbumine in tablets. Chem. Pharm. Bull..

[26] Macedo R.O., do Nascimento T.G., Aragao C.F.S., Gomes A.P.B. (2000). Application of thermal analysis in the characterization of anti-hypertensive drugs. J. Therm. Anal. Calorim..

[27] Ambrozini B., Cervini P., Cavalheiro E.T.G. (2016). Thermal behavior of the β-blocker propranolol. J. Therm. Anal. Calorim..

[28] Silva A.C.M., Galico D.A., Guerra R.B., Perpetuo G.L., Legendre A.O., Rinaldo D., Bannach G. (2015). Thermal stability and thermal decomposition of the antihypertensive drug amlodipine besylate. J. Therm. Anal. Calorim..

[29] Andre V., Cunha-Silva L., Duarte M.T., Santos P.P. (2011). First crystal structures of the antihypertensive drug perindopril erbumine: A novel hydrated form and polymorphs α and β. Cryst. Growth Des..

[30] Budrugeac P., Segal E. (2007). Applicability of the Kissinger equation in thermal analysis. J. Therm. Anal. Calorim..

[31] Ledeti I., Vlase G., Ciucanu I., Olariu T., Fulias A., Suta L.M., Belu I. (2015). Analysis of solid binary systems containing simvastatin. Rev. Chim.-Buchar..

[32] Fulias A., Vlase G., Vlase T., Suta L.M., Soica C., Ledeti I. (2015). Screening and characterization of cocrystal formation between carbamazepine and succinic acid. J. Therm. Anal. Calorim..

[33] Ivan C., Suta L.M., Olariu T., Ledeti I., Vlase G., Vlase T., Olariu S., Matusz P., Fulias A. (2015). Preliminary kinetic study for heterogenous degradation of cholesterol-containing human biliary stones. Rev. Chim.-Buchar..

[34] Fulias A., Soica C., Ledeti I., Vlase T., Vlase G., Suta L.M., Belu I. (2014). Characterization of pharmaceutical acetylsalicylic acid—theophylline cocrystal obtained by slurry method under microwave irradiation. Rev. Chim.-Buchar..

[35] Ilici M., Bercean V., Venter M., Ledeti I., Olariu T., Suta L.M., Fulias A. (2014). Investigations on the thermal-induced degradation of transitional coordination complexes containing (3*H*-2-thioxo-1,3,4-thiadiazol-5-yl)thioacetate moiety. Rev. Chim.-Buchar..

[36] Ledeti I., Vlase G., Vlase T., Suta L.M., Todea A., Fulias A. (2015). Selection of solid-state excipients for simvastatin dosage forms through thermal and nonthermal techniques. J. Therm. Anal. Calorim..

[37] Fulias A., Vlase G., Ledeti I., Suta L.M. (2015). Ketoprofen-cysteine equimolar salt. Synthesis, thermal analysis, PXRD and FTIR spectroscopy investigation. J. Therm. Anal. Calorim..

[38] Ledeti I., Vlase G., Vlase T., Ciucanu I., Olariu T., Todea A., Fulias A., Suta L.M. (2015). Instrumental analysis of potential lovastatin—Excipient interactions in preformulation studies. Rev. Chim.-Bucharest..

[39] Ledeti I., Vlase G., Vlase T., Fulias A. (2015). Kinetic analysis of solid-state degradation of pure pravastatin versus pharmaceutical formulation. J. Therm. Anal. Calorim..

[40] Ledeti I., Vlase G., Vlase T., Fulias A., Suta L.M. (2016). Comparative thermal stability of two similar-structure hypolipidemic agents Simvastatin and Lovastatin-kinetic study. J. Therm. Anal. Calorim..

[41] Ledeti I., Ledeti A., Vlase G., Vlase T., Matusz P., Bercean V., Suta L.M., Piciu D. (2016). Thermal stability of synthetic thyroid hormone l-thyroxine and l-thyroxine sodium salt hydrate both pure and in pharmaceutical formulations. J. Pharm. Biomed..

[42] Ledeti I., Alexa A., Bercean V., Vlase G., Vlase T., Suta L.M., Fulias A. (2015). Synthesis and degradation of schiff bases containing heterocyclic pharmacophore. Int. J. Mol. Sci..

[43] Friedman H.L. (1969). New methods for evaluating kinetic parameters from thermal analysis data. J. Polym. Sci..

[44] Ozawa T. (1965). A new method of analyzing thermogravimetric data. Bull. Chem. Soc. Jpn..

[45] Flynn J.H., Wall L.A. (1966). A quick direct method for determination of activation energy from thermogravimetric data. J. Polym. Sci. B.

[46] Kissinger H.E. (1957). Reaction kinetics in differential thermal analysis. Anal. Chem..

[47] Akahira T., Sunose T. (1971). Joint convention of four electrical institutes. Researvh Report (Chiba Institute Technology). Sci. Technol..

[48] Rucman R., Zupet P. (2006). New Hydrated Crystalline Forms of Perindopril Erbumine, Process for the Preparation thereof and Pharmaceutical Formulations Containing These Compounds. EP Patent App..

